# Rare case report: sclerosing epithelioid fibrosarcoma with FUS-CREB3L1 gene fusion

**DOI:** 10.3389/fonc.2025.1491398

**Published:** 2025-03-12

**Authors:** Tingting Wang, Haimin Xu, Chuanying Li

**Affiliations:** ^1^ Department of Pathology, Lu’an hospital of Anhui Medical University, Anhui, China; ^2^ Department of Pathology, Ruijin Hospital, Shanghai Jiaotong University School of Medicine, Shanghai, China

**Keywords:** sclerosing epithelioid fibrosarcoma (SEF), low-grade fibromyxoid sarcoma (LGFMS), MUC4, FUS-CREB3L1, high-grade transformation (HGT)

## Abstract

Sclerosing epithelioid fibrosarcoma (SEF) is a rare soft tissue malignancy frequently misdiagnosed due to its overlapping immunohistochemical and molecular features with low-grade fibromyxoid sarcoma (LGFMS). We present the case of a 60-year-old male who initially presented with a mass in the left thigh four years ago, which significantly increased in size over the past year. MRI of the femur revealed a large, well-circumscribed mass in the mid and lower left thigh. Surgical excision of the tumor and associated thrombus in the ipsilateral blood vessel was performed. Histomorphological analysis confirmed a pure SEF with no myxoid stroma, aiding in its differentiation from LGFMS. Immunohistochemical staining revealed diffuse and strong MUC4 positivity, while next-generation sequencing (NGS) demonstrated molecular characteristics consistent with LGFMS, specifically FUS-CREB3L1 gene fusion. This case underscores the asynchrony between the pathological morphology and molecular features of soft tissue tumors during their development and differentiation. Although histologically low-grade, SEF typically exhibits a high rate of local recurrence and distant metastasis. We diagnosed this case as SEF and recommended an aggressive clinical treatment regimen. This report aims to raise awareness of the diagnostic challenges associated with SEF and LGFMS.

## Introduction

Sclerosing epithelioid fibrosarcoma (SEF) is a low-grade soft tissue tumor that originates from fibroblasts within tendons, ligaments, and muscle tissue. First described in 1995, SEF most commonly arises in the deep muscle tissue of the lower limbs, though it has also been reported in various other locations, including bone, the maxillofacial region (1), spine (2), kidney (3), and liver (4). SEF predominantly affects middle-aged and elderly individuals, with rare occurrences in children (5). The male-to-female ratio shows no significant difference (6).

Patients often do not experience specific symptoms at the onset, especially when the mass is located in the lower limbs. It is typically discovered only when it enlarges sufficiently to cause local pain or functional impairment. When SEF occurs in visceral organs, it is identified based on the corresponding symptoms or signs. The tumors are usually large at diagnosis, with an average size of around 9 cm (7, 8).

The typical morphological features of SEF include epithelioid tumor cel ls arranged in nests or cords within a collagenous, sclerotic, and hyalinized stroma. SEF shares overlapping features with low-grade fibromyxoid sarcoma (LGFMS) in terms of morphology, immunohistochemical expression, and molecular characteristics, often leading to their classification within the same disease spectrum. The key morphological difference between LGFMS and SEF lies in the tumor cells: LGFMS is characterized by spindle cells with a fibrous stroma and associated mucoid degeneration.

Molecularly, “pure” SEF primarily exhibits EWSR1-CREB3L1 fusion, while most cases of LGFMS show a t(7;16) (q33;p11) translocation resulting in FUS-CREB3L2 fusion, with a few cases showing a t(11;16) (p11;p11) translocation leading to FUS-CREB3L1 fusion (9). Here, we present a unique case of SEF with morphological features consistent with pure SEF and a strongly aggressive biological behavior, yet molecular analysis revealed FUS-CREB3L2 fusion. Given the genetic heterogeneity of this tumor, we reviewed and compared the immunohistochemical and molecular findings reported in the literature to further enhance our understanding of this disease.

## Case presentation

A 60-year-old patient was admitted to the Department of Orthopedics of Shanghai Ruijin Hospital in December 2023, due to the discovery of a mass in the back of the left thigh without tenderness、skin damage and limitation of movement, while pay no attention on the mass during this period. This mass was present for more than four years. But over the past year, it had gradually increased in size.

**Figure 1:**
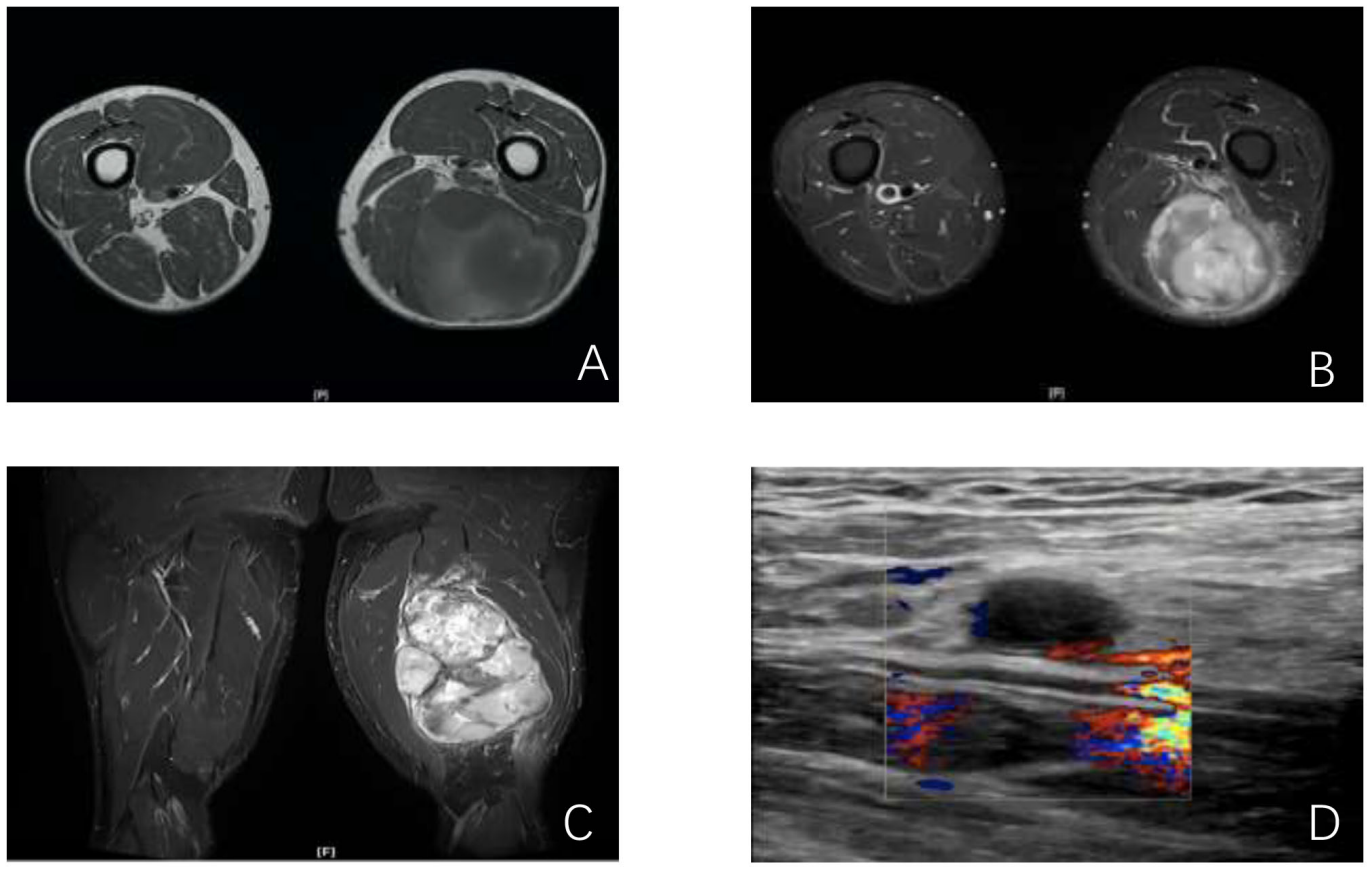
Imaging of the lesions. **(A, B)** MRI cross sections showing the mass in the left thigh; **(C)** Lower-extremity arterial computed tomographic angiography (CTA) longitudinal section showing the positional relationship between the mass and the thigh blood vessels; **(D)** vessel Doppler ultrasonography showing hypoechoic echoes near the left thigh veins.

The patient underwent Magnetic Resonance Imaging (MRI) scan immediately (**Figures 1A, B**). MRI of the femur revealed a large mass in the posterior muscle group of the mid and lower left thigh. The mass measured approximately 14.6 cm × 9.9 cm × 9.3 cm and exhibited heterogeneous signal intensity. On T1-weighted images (T1W), it showed mixed low signal intensity, while on T2-weighted (T2W) and short tau inversion recovery (STIR) images, it showed mixed high signal intensity. The lesion had a general clear boundary, with mild edema observed in the surrounding muscles. No significant abnormal signal was detected in the left femur.

The patient was admitted to our hospital. Throughout the course of the disease, the patient’s body weight, diet, stool, urine, and sleep patterns remained largely unchanged. There was no significant medical history, personal history, or family history. After admission, the patient underwent lower-extremity arterial computed tomographic angiography (CTA) scan of the left thigh (**Figure 1C**). CTA imaging revealed a circular soft tissue mass located in the posterior aspect of the left thigh, involving the biceps femoris, semitendinosus, and semimembranosus muscles. The mass measured approximately 109 mm × 90 mm in size with an upper-to-lower diameter of about 156 mm. The mass had a clear boundary but an irregular edge. CTA shows the positional relationship between the tumor and the thigh blood vessels, as well as the vascular status. Vessel Doppler ultrasonography (**Figure 1D**) showed hypoechoic echoes near the left femoral vein, suggesting abnormal lymph nodes with a size of approximately 27.8mm×8.0mm.

Following completion of the relevant examinations, the tumor and the surrounding intravascular mass in the left thigh were surgically removed and sent for pathological analysis. The operation was successful, and the patient was given routine anti-inflammation, acid inhibition and other rehydration treatment after operation. Keep the wound dry and clean, no blood or fluid seepage. The patient recovered smoothly, and was discharged on postoperative day 7 (POD 7). After discharge, go to outpatient clinic regularly for disinfection and dressing change. The timeline of major patient care is shown in **Table 1**.

**Table 1:** A timeline with relevant data from the episode of care.

Time	Episode of care	Examination and treatment
December 2, 2023	Outpatient service	MRI of the left thigh was performed, Appointment for hospitalization
December 23, 2023	Admitted to hospital	Improved relevant examinations, such as CT 、X-ray、vessel Doppler ultrasonography. et al.
December 27, 2023	Excision surgery of the left leg mass	Pathological biopsy of the tumor
January 2, 2024	Postoperative examination	The surgical area was monitored by X-ray
January 4, 2024	Discharged from hospital	Regular disinfection and dressing changes were performed in the outpatient clinic, followed by a follow-up visit and review by our orthopedic specialists at our hospital one month later.
March 4, 2024	Orthopedic specialist outpatient follow-up	Recommended additional radiotherapy
After March 4, 2024	Radiotherapy	Radiotherapy 6 times in other hospital, the specific program is unknown.

Resection of the soft tissue lesion of the left thigh was performed. The pathologist received a surgical specimen of the tumor that was described as follows: a mass attached to the skin, with a total size of 22.0 ×14.0 ×10.0cm. The skin is smooth and with an area of 10.0 ×3.5cm. An underlying muscle tissue is of 18.0 ×9.0 ×7.0cm, and a mass of 15.0 ×12.0 ×8.0cm adjacent to the muscle. Part of the cut surface of the mass is gray and delicate, and part of the mass is tough.

Histopathological examination revealed that the tumor had infiltrated the striated muscle. The tumor cells were medium-sized, oval, and exhibited epithelioid characteristics. They were arranged in cords or sheets within a significantly sclerotic stroma composed of hyaline-degenerated, red-stained collagen fibers, with localized ossification and calcium salt deposition. The tumor margins were negative.

**Figure 2:**
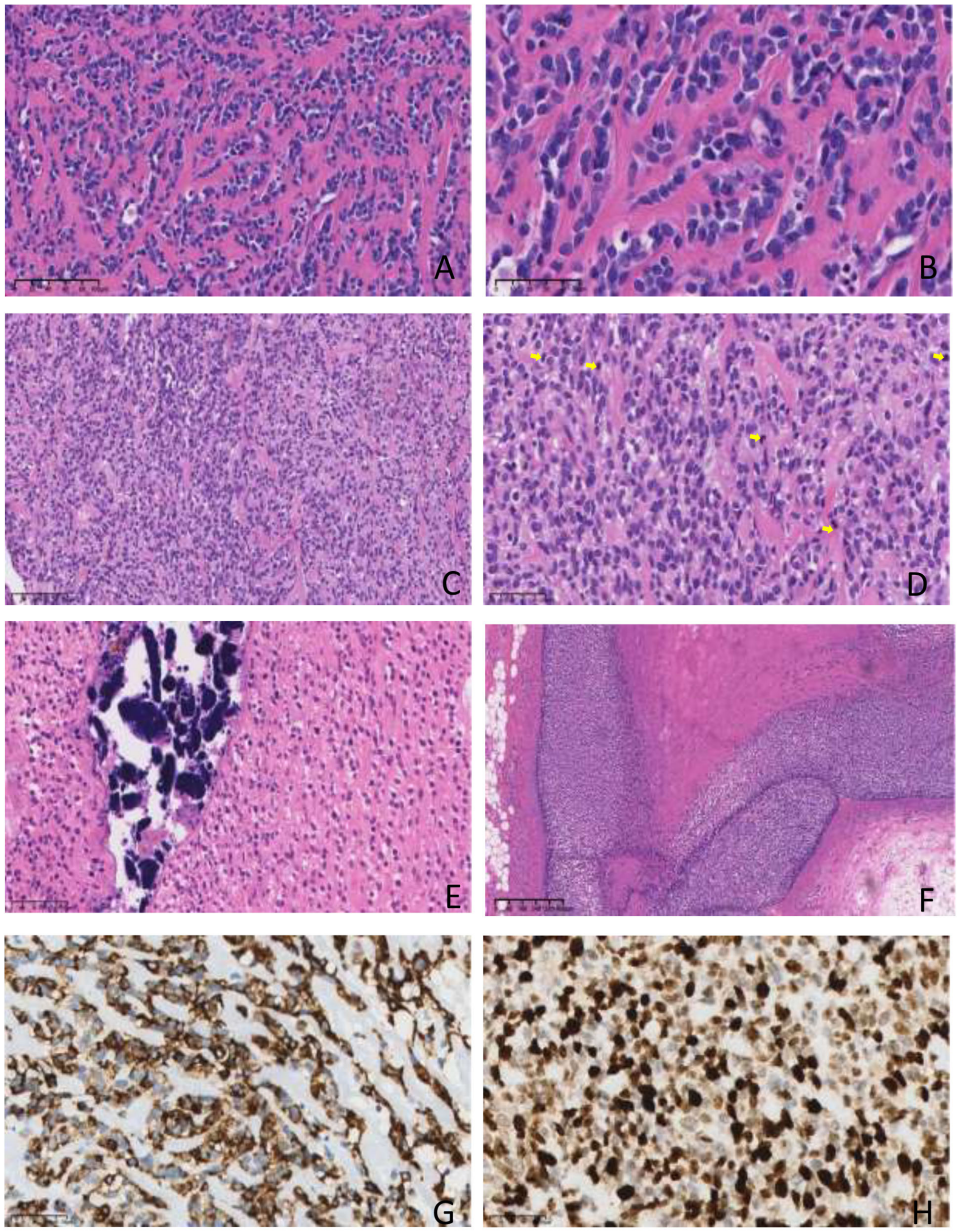
Hematoxylin and eosin (HE) staining and immunohistochemical (IHC) staining showing tumor tissue morphology and immunophenotype. **(A, B)** HE showing the classical morphology of SEF, revealed epithelioid and spindle-shaped tumor cells arranged in thread-like and nest-like patterns within a highly sclerotic (**A**. Scale bar, 100 µM, **B**. Scale bar, 50 µM). **(C, D)** HE showing a local area with significantly increased cell density, crowded arrangement. Cellular atypia was prominent, and mitotic figures were easily found (A. Scale bar, 100 µM, **(B)** Scale bar, 50 µM, yellow arrows indicating cells with nuclear divisions in the tumor). **(E)** Local calcification was observed(Scale bar, 50 µM). **(F)** Showing local necrosis of the tumor(Scale bar, 400 µM). **(G, H)** IHC showing strong positive expression of MUC4 and high expression of Ki67 (Scale bar, 50 µM).

The morphological features were consistent with SEF within the spectrum of soft tissue sarcomas. To confirm the diagnosis, we performed immunohistochemical staining with a series of markers. The tumor cells showed positive expression for Vimentin, ERG, MUC4, TLE1, CD99, CD56, Ki67 (approximately 80%), and H3K27Me3. Other markers were negative, including Desmin, S-100, SMA, Calponin, MyoD1, BCOR, FOSB, FOS, SATB-2, H3K27M, CD3, CD20, SYN, Melan A, SOX10, NKX2.2, MDM2, CDK4, AE1/AE3, TFE-3, WT-1, PAX-8, CD34, and CD31. The strong positive expression of MUC4 and Vimentin, along with the elevated Ki67 index, was indicative of high tumor reactivity (**Figure 2**).

**Figure 3:**
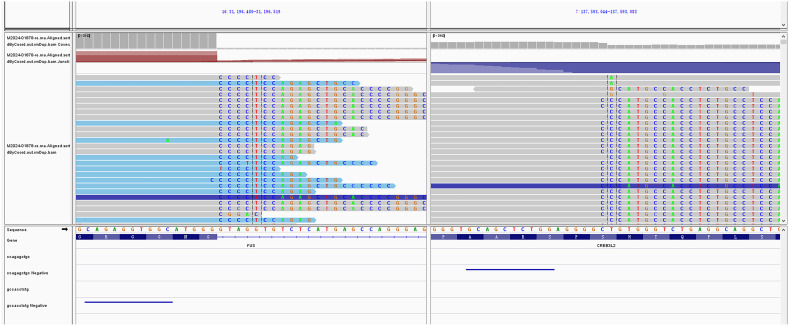
Molecular characterization of the tumor. Next-generation sequencing (NGS) of the thigh mass revealed the FUS::CREB3L2 gene fusion.

Given the rarity of SEF, we proceeded with molecular testing to further support the diagnosis. Next-generation sequencing (NGS) of RNA and DNA from the left thigh mass was performed, targeting 285 and 654 cancer-related gene mutation sites, respectively. The analysis identified FUS::CREB3L2 as the fusion gene, which is of clear clinical significance (**Figure 3**).

Two months after discharge, the patient went to the expert clinic for follow-up, and returned to the local area for radiotherapy under the guidance of the experts in our hospital. At the 10-month postoperative follow-up, the patient had completed 6 courses of radiotherapy. However, the specific treatment plan is unknown. The patient reported no significant complications and no signs of tumor recurrence, and is currently in good condition.

## Discussion

The World Health Organization classifies sclerosing epithelioid fibrosarcoma (SEF) as a malignant fibroblastic/myofibroblastic tumor with low-grade malignancy. In this report, we present a unique case featuring pure SEF morphology alongside molecular characteristics typically associated with low-grade fibromyxoid sarcoma (LGFMS). We speculate that this phenotype may reflect a high-grade transformation or dedifferentiation process in soft tissue tumors. SEF and LGFMS are considered to belong to the same disease spectrum (2), likely due to their similar immunohistochemical features, such as Vimentin and cytoplasmic MUC4 expression and comparable molecular alterations. However, these tumors exhibit distinct HE morphologies and biological behaviors, making accurate differential diagnosis critical.

Several studies have demonstrated that MUC4 has high sensitivity in detecting both SEF and LGFMS (10, 11). MUC4 is a transmembrane mucin and serves as the membrane ligand for the ErbB2 receptor tyrosine kinase. Abnormal expression of MUC4 can disrupt epithelial cell polarity and promote epithelial-mesenchymal transition (EMT), thereby enhancing cellular motility and invasion capabilities (12).

Both SEF and LGFMS are associated with chromosomal translocations and chimeric fusions, sharing similar translocation patterns. The most common SEF translocation is EWSR1::CREB3L1, accounting for approximately 60% of cases, or the exchange of PAX5 and/or CREB3L1 with CREB3L2, CREB3L3, or CREM. FUS rearrangement is a hallmark of LGFMS/mixed fibromyxoid fibrosarcoma, showing the FUS-CREB3L2 chimera (13, 14), which is rare in “pure” SEF (15), occurring in only about 9% of cases. Despite these molecular similarities, SEF and LGFMS differ significantly in their natural history, clinical presentation, and prognosis. SEF is more aggressive than LGFMS (13, 16), with a higher risk of metastasis and recurrence (17). Mortality is slightly higher in patients with SEF alone (44%) compared to those with mixed SEF/LGFMS tumors (37%) (18).

In this particular case, the tumor originated in the lower limb and thigh—common sites for SEF—and was associated with a tumor thrombus in the ipsilateral thigh vessel at presentation. After thorough sampling and evaluation by experienced pathologists, the tumor cells were found to be small to medium in size, arranged in epithelioid nests, with stromal hyalinization and collagenization. This case displayed no myxoid matrix and lacked LGFMS morphological characteristics, aligning more closely with “pure” SEF.

The tumor was large (14.6 cm × 9.9 cm × 9.3 cm) with a Ki-67 index of 80%. The mitotic rate ranged from 2 to 7 per 10 high-power fields (mean: 4/10 HPFs). Necrosis is reported in about half of SEF cases (18), the present case did have areas of necrosis. Notably, the tumor exhibited intravascular thrombus formation at the same site. Next-generation sequencing (NGS) confirmed the presence of the FUS-CREB3L1 fusion gene, consistent with LGFMS. However, based on the tumor’s microscopic morphology and biological behavior, a diagnosis of pure SEF was deemed more appropriate.

We hypothesize that this distinctive morphology and molecular phenotype may be linked to the high-grade transformation (HGT) process of low-grade malignant soft tissue sarcoma, where tumor development is driven by the FUS:: CREB3L2 fusion, leading to dedifferentiation into a pure SEF form. Recently, Tay et al. described an LGFMS case with dedifferentiation (19), where the tumor exhibited both LGFMS and SEF histology, with a sudden transition between the two. Immunohistochemical analysis of both morphologies showed MUC4 positivity, and FISH testing confirmed FUS translocation, with the molecular test revealing FUS-CREB3L2 fusion in regions with high-grade morphology. The progression of LGFMS, from the primary lung tumor to metastasis in the pancreas and mediastinum, was marked by SEF morphology and FUS::CREB3L2 fusion (20). Dedifferentiation was linked to shorter survival periods (21).

Though SEF patients face a greater risk of invasion, metastasis, and recurrence, there are no unambiguous guidelines for treatment strategies. Surgical resection with negative margins remains the primary treatment, and postoperative radiotherapy and chemotherapy may help control tumor recurrence and metastasis (22). In this case, the tumor was extensively resected. During the most recent follow-up, we learned that the patient came to our specialist clinic to receive treatment advice on additional radiotherapy. The patient received six radiation treatments and showed no signs of local or distant recurrence at ten months follow-up. There are no signs of recurrence or metastasis. Long-term follow-up will further evaluate the patient’s prognosis. A retrospective cohort studies suggest that providing palliative chemotherapy to patients with SEF may benefit a minority of patients (23). This may be due to tumor heterogeneity and individual differences (6). Checkpoint inhibitors are still under investigation in soft tissue sarcoma (24). A retrospective study of 80 patients (LGFMS, SEF, H-LGFMS/SEF) treated with medical agents, the results show that Pazoparib appears to be the most active agent in LGFMS and SEF and immune checkpoint inhibitors prolong disease stability (25). However, immunotherapy is administered simultaneously with concurrent radiotherapy or chemotherapy, and it is difficult to assess whether immunotherapy is the driver of the improved clinical response. Immunotherapy may be a promising treatment route for SEF (16), needs to be confirmed in larger case studies. We will continue to pay attention on the relationship between tumor HE morphology, molecular phenotype, treatment strategies and disease prognosis in the SEF-LGFMS spectrum.

## Data Availability

The datasets presented in this study can be found in online repositories. The names of the repository/repositories and accession number(s) can be found in the article/supplementary material.
